# Evolutionary diversity of proton and water channels on the oxidizing side of photosystem II and their relevance to function

**DOI:** 10.1007/s11120-023-01018-w

**Published:** 2023-06-02

**Authors:** Rana Hussein, Mohamed Ibrahim, Asmit Bhowmick, Philipp S. Simon, Isabel Bogacz, Margaret D. Doyle, Holger Dobbek, Athina Zouni, Johannes Messinger, Vittal K. Yachandra, Jan F. Kern, Junko Yano

**Affiliations:** 1 Department of Biology, Humboldt-Universität Zu Berlin, 10099 Berlin, Germany; 2 Molecular Biophysics and Integrated Bioimaging Division, Lawrence Berkeley National Laboratory, Berkeley, CA 94720 USA; 3 Molecular Biomimetics, Department of Chemistry-Ångström, Uppsala University, SE 75120 Uppsala, Sweden; 4 Department of Chemistry, Umeå University, SE 90187 Umeå, Sweden

**Keywords:** Photosystem II, Water oxidation, Water transport, Oxygen evolving complex, Evolution

## Abstract

One of the reasons for the high efficiency and selectivity of biological catalysts arise from their ability to control the pathways of substrates and products using protein channels, and by modulating the transport in the channels using the interaction with the protein residues and the water/hydrogen-bonding network. This process is clearly demonstrated in Photosystem II (PS II), where its light-driven water oxidation reaction catalyzed by the Mn_4_CaO_5_ cluster occurs deep inside the protein complex and thus requires the transport of two water molecules to and four protons from the metal center to the bulk water. Based on the recent advances in structural studies of PS II from X-ray crystallography and cryo-electron microscopy, in this review we compare the channels that have been proposed to facilitate this mass transport in cyanobacteria, red and green algae, diatoms, and higher plants. The three major channels (O1, O4, and Cl1 channels) are present in all species investigated; however, some differences exist in the reported structures that arise from the different composition and arrangement of membrane extrinsic subunits between the species. Among the three channels, the Cl1 channel, including the proton gate, is the most conserved among all photosynthetic species. We also found at least one branch for the O1 channel in all organisms, extending all the way from Ca/O1 via the ‘water wheel’ to the lumen. However, the extending path after the water wheel varies between most species. The O4 channel is, like the Cl1 channel, highly conserved among all species while having different orientations at the end of the path near the bulk. The comparison suggests that the previously proposed functionality of the channels in *T. vestitus* (Ibrahim et al., Proc Natl Acad Sci USA 117:12624–12635, 2020; Hussein et al., Nat Commun 12:6531, 2021) is conserved through the species, i.e. the O1-like channel is used for substrate water intake, and the tighter Cl1 and O4 channels for proton release. The comparison does not eliminate the potential role of O4 channel as a water intake channel. However, the highly ordered hydrogen-bonded water wire connected to the Mn_4_CaO_5_ cluster via the O4 may strongly suggest that it functions in proton release, especially during the S_0_ → S_1_ transition (Saito et al., Nat Commun 6:8488, 2015; Kern et al., Nature 563:421–425, 2018; Ibrahim et al., Proc Natl Acad Sci USA 117:12624–12635, 2020; Sakashita et al., Phys Chem Chem Phys 22:15831–15841, 2020; Hussein et al., Nat Commun 12:6531, 2021).

## Introduction

**Fig. 1:**
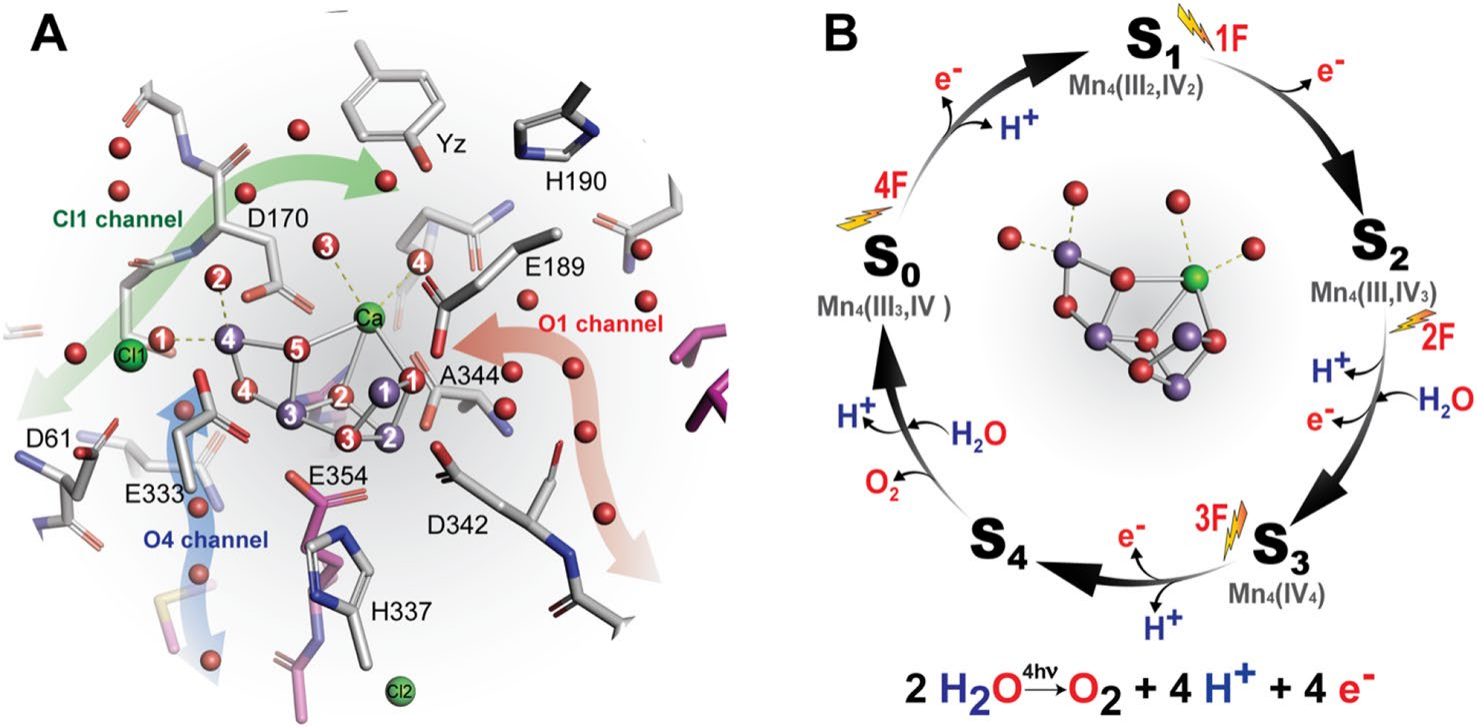
The catalytic site of water oxidation in PS II. **A** The structure of the Mn_4_CaO_5_ cluster in the S_1_ state with its surrounding ligands. The water channels connecting the Mn_4_CaO_5_ cluster to the lumen (O1, O4, and Cl1) are indicated as red, blue, and green shaded arrows, respectively. **B** Kok cycle showing the steps of water oxidation reaction triggered by the absorption of photons shown as four light flashes (1F to 4F). The oxidation state of the Mn_4_CaO_5_ cluster is denoted by the S_i_ states (S_0_–S_4_). The cycle shows the steps of electron (e^−^) and proton (H^+^) release and the insertion of two water molecules (H_2_O). Labels: Mn purple, Ca^2+^ green, O red. Amino acid residues from D1 and CP43 subunits are colored gray and magenta, respectively

In nature, the light-driven water oxidation reaction is carried out by Photosystem II (PS II), a multisubunit protein complex that is part of the thylakoid membrane in chloroplasts or cyanobacterial cells. This reaction is made possible through a series of spatially separated cofactors that extend over 40 Å and include the metal catalytic center (Oxygen Evolving Complex, OEC), the redox-active tyrosine Y_Z_, the reaction center chlorophylls, pheophytin, and two plastoquinone electron acceptors (Q_A_ and Q_B_) (Hillier and Messinger 2005; Müh and Zouni 2020; Shevela et al. 2021). The water oxidation reaction (shown below) proceeds by coupling the one-electron photochemistry occurring at the reaction center chlorophylls with the two-electron, two-proton reductive chemistry at the Q_B_ site and the four-electron, four-proton oxidation of water at the OEC, that consists of a heteronuclear Mn_4_CaO_5_ cluster (Fig. 1A) (Umena et al. 2011; Yano and Yachandra 2014).

$$2{\text{H}}_{2} {\text{O}} \to {\text{O}}_{2} + 4{\text{H}}^{ + } + 4{\text{e}}^{ - }$$

During water oxidation catalysis, the OEC cycles through five intermediate redox states, the S-states (S_0_ to S_4_) (Fig. 1B)(Kok et al. 1970; Joliot and Kok 1975), that correspond to the successive light-driven abstraction of four electrons and four protons from the OEC, as well as the uptake of two water molecules. Starting from the lowest oxidation state (S_0_), the first three electron abstractions each lead to the oxidation of one Mn(III) to a Mn(IV), while the S_4_ state likely represents the formation of a transient oxidized Mn(V) or Mn(IV) with a bound delocalized oxyl radical that is highly reactive and initiates O_2_ formation with a specific oxygen ligand (Cox and Messinger 2013; Yano and Yachandra 2014; Vinyard and Brudvig 2017; Yamaguchi et al. 2022). Substrate water is incorporated into the Mn_4_CaO_5_ cluster in two separate steps: one is introduced during the S_2_ → S_3_ transition and the second during the S_3_ → S_0_ transition (Noguchi and Sugiura 2002; Suzuki et al. 2008; Cox and Messinger 2013; Suga et al. 2017; Kern et al. 2018). Meanwhile, four protons are released from the catalytic reaction into the lumen, in the pattern of 1:0:1:2 for the S-state transitions S_0_ → S_1_ → S_2_ → S_3_ → [S_4_] → S_0_, respectively (Renger 2012; Noguchi et al. 2012; Cox and Messinger 2013; Klauss et al. 2015) (Fig. 1B). Once four oxidizing equivalents accumulate at the OEC (reactive [S_4_]-state), the release of O_2_ and the formation of the S_0_-state take place spontaneously.

Throughout this process, the spatially controlled transport of the substrate, water, and one of the products, protons, between the catalytic center and the inside of the thylakoids (lumen) is essential for catalytic efficiency. The O_2_ release pathway(s), if any, are not well established experimentally at present, and it is thought to be most likely a non-specific diffusion through the hydrophobic peptide and lipid matrix (Gabdulkhakov et al. 2009, 2015a).

There have been a number of theoretical studies (Murray and Barber 2007a, b; Ho and Styring 2008; Ho 2008; Gabdulkhakov et al. 2009, 2015a; Vassiliev et al. 2010, 2012) to identify the channels that facilitate substrate intake and proton release, based on the crystallographic data obtained from PS II of Thermosynechococcus bacteria, *T. vestitus* (previously known as *T. elongatus*, see (Komárek et al. 2020)) and *T. vulcanus* (Ferreira et al. 2004; Loll et al. 2005; Guskov et al. 2009; Gabdulkhakov et al. 2009; Umena et al. 2011). Three-branched channel systems were identified and variously named (Table 1). Here we employ the nomenclature that signifies the entry point of each channel into the OEC region: O1 channel, O4 channel, and Cl1 channel (see Fig. 1A).

**Table 1:** Nomenclature of the channels in literature

Study	Ho and Styring (Ho and Styring 2008)	Murray and Barber (Murray and Barber 2007a)	Gabdulkhakov et al. (Gabdulkhakov et al. 2009)	Umena et al. (Umena et al. 2011)	Vassiliev et al. (Vassiliev et al. 2012)	Ogata et al. (Ogata et al. 2013)	Sakashita et al. (Sakashita et al. 2017b)	Weisz et al. (Weisz et al. 2017)
Method	Surface contact calculation	CAVER	Xe, CAVER	H-bond network analysis	MD simulation			Mass spectrometry
PDB ID	2AXT	1S5L and 2AXT	3BZ1	3ARC (2WU2)	3ARC (2WU2)	3ARC (2WU2)	3ARC (2WU2)	
Resolution	3.00 Å	3.5 Å, 3.0 Å	2.90 Å	1.90 Å	1.90 Å	1.90 Å	1.90 Å	
O1 Channel A	large	channel ii	B1		4.A		O1-water chain	Arm 2
O1 Channel B	large	channel ii	B2		4B			Arm 2
O4 Channel	narrow	NA	E, F	4.c	2	Path 3	O4-water chain	
			D		Channel X			Arm 3
Cl1 Channel A	broad	*-*	G	4.b	1		E65/E312 channel	
Cl1 Channel B		channel iii	C, D		3	Path 2		Arm 1
Cl2 network				4.c				
	back	channel i	A1, A2	3.b	5	Path 1		Arm 3

There are multiple names used for identifying the water and proton channels in PS II. The table summarizes their correspondence

Recent structural studies propose that the O1 channel is likely the main water intake pathway (Suga et al. 2019; Ibrahim et al. 2020; Li et al. 2021; Hussein et al. 2021). This hypothesis is based on the high mobility of waters located along the O1 channel compared to the other channels. On the other hand, as W20 (named as W665 (Suga et al. 2019)) is absent starting in the S_2_ state the H-bonding network for proton release is disrupted, making a possible proton release via the O4 channel during the S_2_ → S_3_ → S_0_ transitions unlikely (Kern et al. 2018; Suga et al. 2019). Therefore, the Cl1 channel is likely the main proton release pathway, at least during the S_2_ → S_3_ and the S_3_ → S_0_ transition. This is indicated, among others, by the reversible rotation of the amino acid residue D1-E65, which likely opens a gate for proton release into the bulk (Hussein et al. 2021). Recent experimental and computational studies also suggest that the O4 channel is used for proton transfer during the S_0_ → S_1_ transition (Saito et al. 2015; Takaoka et al. 2016; Shimizu et al. 2018). These results indicate that the water oxidation reaction at the OEC is well synchronized with the movement of specific amino acid side chains and the hydrogen-bonding network over the entire length of the channels, which is essential for shuttling substrate waters and protons.

Despite the long evolutionary separation, it is well established that the PS II reaction center structure is overall highly conserved among oxygenic organisms ranging from cyanobacteria and algae to higher plants. However, some differences exist among the organisms in the composition of extrinsic protein subunits located at the lumenal side of the protein and in the associated light-harvesting complexes (Müh and Zouni 2020). Such differences could influence the structure of potential channels, thereby affecting the access of substrate waters to or the release of protons from the OEC (de Lichtenberg et al. 2021).

In this review, we present a comparative study of the channels in the PS II of different species to elucidate the essential components regulating proton egress and water access to the catalytic site. Taking advantage of the recent progress in the structural studies of PS II using serial crystallography and cryo-EM, we investigate here the location and the structure of channels within the PS II complex among different organisms. This structural comparison provides insight into the essential requirements for the regulation of the transport of substrate and protons. Furthermore, it shows the development of different organisms, from cyanobacteria to higher plants, during evolution.

## The architecture of the lumenal side of PS II in different organisms

**Fig. 2:**
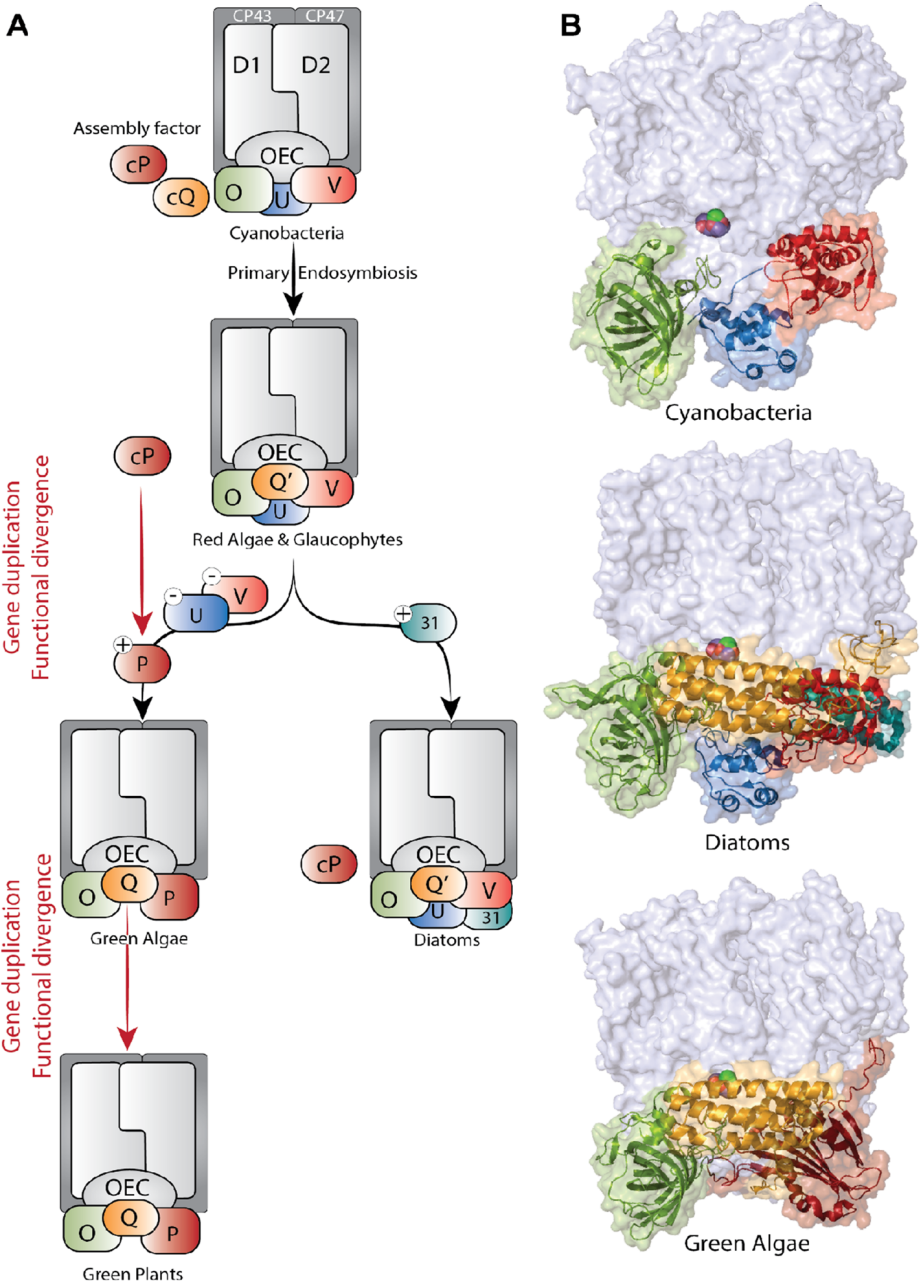
Extrinsic subunits in oxygenic photosynthetic organisms. **A** schematic overview of the extrinsic subunits evolution in PS II. **B** Side view of the monomeric PS II highlighting the structure of the extrinsic subunits in cyanobacteria (PDB Id:7RF2), diatoms (PDB Id:6JLU), and green algae (PDB Id:6KAC)

Photosystem II is a multiprotein complex comprised of several membrane-intrinsic- and membrane-extrinsic subunits. Extrinsic subunits face the lumenal side of the PS II complex and play a role in stabilizing and protecting the Mn_4_CaO_5_ cluster from the lumenal surface. Moreover, they enhance the availability of the inorganic cofactors i.e., Ca^2+^ and Cl^−^ (Ghanotakis et al. 1984), and control the accessibility of the water channels that supply the Mn_4_CaO_5_ cluster with the substrate waters or act as an exit channel for protons and molecular oxygen to guarantee efficient turnover for PS II. Most of the membrane intrinsic subunits are ubiquitous and conserved across all the oxygenic photosynthetic organisms, including cyanobacteria, algae, and higher plants. However, the extrinsic subunits exhibit significant differences except for the PsbO subunit, which showed high similarity and is being conserved among the oxygenic photosynthetic organisms (Roose et al. 2016) (Fig. 2). This emphasizes the importance of the PsbO subunit as a manganese-stabilizing protein. Futhermore, several studies suggest that the PsbO subunit plays a role in proton release (De Las Rivas and Barber 2004; Popelkova and Yocum 2011; Ifuku 2015). Prior to the comparison of potential channels, we thus summarize the differences of the extrinsic subunit composition in different organisms, in relation to their phylogeny.

In cyanobacteria, the extrinsic subunits consist of PsbO, PsbU, PsbV, CyanoQ, and CyanoP (Roose et al. 2016; Ifuku and Nagao 2021). The currently available X-ray and cryo-EM structures of the thermophilic cyanobacterial PS II show the binding of PsbO, PsbU, and PsbV, whereas the binding of CyanoQ or CyanoP is not observed. Interestingly, the recent cryo-EM structure of PS II from the mesophilic cyanobacterium, *Synechocystis PCC 6803*, showed the binding of CyanoQ (Gisriel et al. 2022). It was also reported that CyanoQ is part of PS II assembly intermediates (Michoux et al. 2014). Similarly, for CyanoP, several studies showed that it is part of the premature PS II, suggesting that it may act as an assembly factor in the early stages of the PS II assembly (Selao et al. 2016).

The first photosynthetic eukaryotes developed by endosymbiosis involving an early cyanobacteria taken up by a protist. These then developed further into various branches, such as the glaucophyte, rhodophyte algae (red algae), diatoms, green algae, and plants. In the glaucophyte and the red lineage photosynthetic organisms, including red algae and diatoms, the PsbV and PsbU subunits are highly conserved. In addition, they developed PsbQ’ from its homolog CyanoQ. In contrast, the Psb31 subunit is a unique extrinsic subunit found only in PS II of diatoms.

In the green photosynthetic organisms, including green algae and higher plants, the PsbQ subunit is found which is structurally highly similar to the PsbQ’ in the red lineage but with the presence of further functional deviations. The major difference in the green lineage compared to the red lineage was the replacement of the PsbV and PsbU subunits by the PsbP subunit, that evolved from CyanoP (see (Ifuku and Nagao 2021) for a review).

## Structural studies of PS II from different organisms

High-resolution structures of mature PS II have been obtained by X-ray crystallography at both cryogenic (100 K) (Umena et al. 2011; Hellmich et al. 2014; Suga et al. 2015, 2019; Tanaka et al. 2017) and room temperature (Young et al. 2016; Suga et al. 2017; Kern et al. 2018; Ibrahim et al. 2020; Hussein et al. 2021), and by cryo-electron microscopy (Kato et al. 2021; Gisriel et al. 2022). Therefore, we first provide a brief summary of the recent structural studies (Table 2).

**Table 2:** Summary of the high-resolution PS II structures

PDB ID	Organism	Data collection	Resolution	No. of waters	Reference
**7RF2**	***T. vestitus BP-1***	**RT-XFEL**	**2.08**	**1994**	Hussein et al. (2021**)**
7RF1	*T. vestitus BP-1*	RT-XFEL	1.89	1960	Hussein et al. (2021)
6W1O	*T. vestitus BP-1*	RT-XFEL	2.08	2037	Ibrahim et al. (2020)
6DHE	*T. vestitus BP-1*	RT-XFEL	2.05	2021	Kern et al. (2018)
4PJ0	*T. vestitus BP-1*	cryo-X-ray	2.44	304	Hellmich et al. (2014)
5MX2	*T. vestitus BP-1* (apo-PSII)	cryo-X-ray	2.55	703	Zhang et al. (2017)
4V82	*T. vestitus BP-1* (PSII with terbutryn)	cryo-X-ray	3.2	0	Broser et al. (2011)
**7D1T**	***T. vulcanus***	**cryo-EM**	**1.95**	**2432**	Kato et al. (2021**)**
7COU	*T. vulcanus*	RT-XFEL	2.25	2118	Li et al. (2021)
6JLJ	*T. vulcanus*	cryo-XFEL	2.15	2616	Suga et al. (2019)
5WS5	*T. vulcanus*	RT-XFEL	2.35	1626	Suga et al. (2017)
5B5E	*T. vulcanus*	cryo-X-ray	1.87	3802	Tanaka et al. (2017)
5B66	*T. vulcanus*	cryo-X-ray	1.85	4066	Tanaka et al. (2017)
4UB6	*T. vulcanus*	cryo-XFEL	1.95	2590	Suga et al. (2015)
3WU2	*T. vulcanus*	cryo-X-ray	1.9	2983	Umena et al. (2011)
7YQ2	*T. vulcanus* (PsbA2)	cryo-X-ray	1.9	3468	Nakajima et al. (2022)
7YQ7	*T. vulcanus* (PsbA3)	cryo-X-ray	1.90	3409	Nakajima et al. (2022)
**7N8O**	***S.* sp. PCC 6803**	**cryo-EM**	**1.93**	**1236**	Gisriel et al. (2022**)**
**4YUU**	***C. caldarium* (red algae)**	**cryo-X-ray**	**2.77**	**18**	Ago et al. (2016**)**
**6JLU**	***C. gracilis* (Diatom)**	**cryo-EM**	**3.02**	**1**	Pi et al. (2019**)**
**6KAC**	***C. reinhardtii* (green algae)**	**cryo-EM**	**2.7**	**595**	Sheng et al. (2019**)**
**5XNL**	***P. sativum***	**cryo-EM**	**2.7**	**1076**	Su et al. (2017**)**
**3JCU**	***S.oleracea***	**cryo-EM**	**3.2**	**0**	Wei et al. (2016**)**
5MDX	*A. thaliana* (apo-PSII)	cryo-EM	5.3	0	van Bezouwen et al. (2017)
7OUI	*A. thaliana* (apo-PSII)	cryo-EM	2.79	139	Graca et al. (2021)

The structures analyzed by CAVER 3.0 (Chovancova et al. 2012) in this study are highlighted in bold

### X-ray crystallography

Over the last two decades, X-ray crystallography of PS II using thermophilic cyanobacteria (*Thermosynechococcus vestitus* and *Thermosynechococcus vulcanus*) has revealed the complex protein/cofactor/lipid assembly. There have been two approaches to the structural study; one is to further improve the resolution of the dark resting state, and the other is to follow the structure of the catalytic intermediates under functional conditions. The former effort using low-X-ray dose experiments at cryogenic temperature using synchrotrons has improved the resolution to 1.85 Å (Tanaka et al. 2017). The later approach at room temperature (RT) became possible owing to the introduction of the X-ray free electron lasers (XFELs) like the LINAC Coherent Light Source (LCLS) in the US and the Sub-Ångstrom Compact LAser (SACLA) in Japan (Suga et al. 2017; Kern et al. 2018; Ibrahim et al. 2020). Both approaches at synchrotrons and XFELs described above provide avenues to overcome or moderate the problem of radiation-induced changes to the metal cluster in PS II during traditional X-ray crystallographic data collection (Yano et al. 2005; Kern et al. 2013; Fransson et al. 2021).

Crystallography at XFELs, in particular, provided the ability to take snapshots of the structure at the various time points during the reaction, and provided the structures of all the stable intermediates (S_0_ to S_3_) at room temperature (Kern et al. 2018; Ibrahim et al. 2020). It also allows for the investigation of water movements and changes in hydrogen bonding networks (Ibrahim et al. 2020; Hussein et al. 2021), which can lead to identifying water and proton pathways. PS II reaction centers are fully active in the crystalline environment under the physiological temperatures that are used during the experiments, as evidenced by the ability to advance the reaction through the Kok cycle in situ, shown by the crystallography and X-ray spectroscopy results (Fransson et al. 2018, 2021; Kern et al. 2018; Ibrahim et al. 2020) as well as supplementary spectroscopic and mass spectrometric measurements on microcrystals of PS II (Kato et al. 2018, 2019; Kern et al. 2018; Ibrahim et al. 2020).

Besides *Thermosynechococcus* species, a crystal structure of eukaryotic PS II from red alga (*Cyanidium caldarium*) has been reported at 2.76 Å resolution (Ago et al. 2016). The major difference to the *Thermosynechococcus* PS II structure is the presence of the PsbQ’ subunit at the lumenal side of CP43, that contributes to the difference of channels.

### Cryo electron microscopy

Another recent advancement in structural studies arises from the application of cryo-electron microscopy. Unlike the X-ray structural studies that require high-quality crystals, which is a highly elaborate task for large membrane proteins such as PS II, cryo-EM can provide single particle images of PS II (Kato et al. 2021; Zabret et al. 2021; Gisriel et al. 2022). The method allows obtaining structures of PS II from species other than of the *Thermosynechococcus* family that have been difficult to crystallize for structural studies with diffraction methods, often due to the highly variable light harvesting complexes associated with PS II (Pi et al. 2019; Sheng et al. 2019; Graca et al. 2021).

The cryo-EM structure of PS II from the mesophilic cyanobacterium *Synechocystis* sp. PCC 6803 (Gisriel et al. 2022) has been reported at a resolution of 1.93 Å. A number of differences are observed relative to thermophilic PS II structures, that include the presence of extrinsic subunit CyanoQ, the flexibility of the C terminus of the D1 subunit, and differences in the PsbV subunit that affect the path for the Large (O1) water channel. As reported by Kato et al*.*, however, electron beam damage, in particular around the redox-active regions like the OEC, at the high-dose utilized to obtain high-resolution data has been a challenge that needs to be solved for cryo-EM studies (Kato et al. 2021).

When attempting to extract structural diversity that originates from the intrinsic differences among the species from the above experiments, one needs to pay attention to the potentially convoluted effects that arise from the methods and experimental conditions. Parameters that differ among experiments include temperature, radiation dose, and the form of samples. The cryo-EM and synchrotron X-ray crystallography experiments are carried out at a cryogenic temperature, while X-ray crystallography at XFELs is mostly performed at room temperature (Kern et al. 2013, 2018; Young et al. 2016; Suga et al. 2017). Radiation-induced changes in proteins and redox-active metal centers could occur to various degrees depending on the radiation (electrons or X-rays) dose. While radiation-induced sample damage that arises from the diffusion of radicals and solvated electrons is an intrinsic problem in cryo-EM and synchrotron X-ray diffraction experiments (Yano et al. 2005; Holton 2009; Kato et al. 2021; Garman and Weik 2021), the femtosecond X-ray pulses utilized in XFEL diffraction experiments allow the use of a higher radiation dose even at room temperature without manifestation of the damage effect in the measured data due to the “measure before destroy” approach (Neutze et al. 2000; Kern et al. 2013; Chapman 2019; Fransson et al. 2021). The issue of the form of samples (single particles, crystals, etc.) is more complicated due to the different sample treatments prior to the experiments. It can affect the stability of the Mn_4_CaO_5_ cluster and natural or artificial electron acceptor (e.g., quinones) contents. Changes in the cluster stability can cause a reduction of Mn(III/IV) to Mn(II) and disassembly of the cluster, which changes the position/orientation of amino acids and waters near the cluster. Changes in the acceptor content can inhibit the S-state advancement of the catalytic center. Checking the O_2_ activity of each sample prior to the structural study under conditions as close to the structural measurements as possible is therefore important. For example, PS II crystals are as active as solution samples (Zouni et al. 2000; Krivanek et al. 2007; Kern et al. 2018; Ibrahim et al. 2020), although slight differences in the O_2_ evolution have been observed (Kato et al. 2018, 2019) that could be due to increased concentrations of cryoprotectants, e.g., glycerol.

It should be noted that for several high-resolution crystal structures, a so-called “dehydration” (Umena et al. 2011; Tanaka et al. 2017) or “post-crystalization treatment” (Hellmich et al. 2014; Kern et al. 2018; Ibrahim et al. 2020; Hussein et al. 2021) procedure was applied. Generally, the actual mode of action of the post-crystalization treatment in the case of PS II crystals sometimes includes a transition into a more membrane-like environment during the procedure (Hellmich et al. 2014). In that case, several observations support the occurrence of this transition: (a) the polyethylene glycol mono methyl ether utilized in the dehydration procedure is known to provide a more membrane or detergent-like environment for hydrophobic proteins compared to normal polyethylene glycol (Brzozowski and Tolley 1994). (b) the crystal packing after the “post-crystalization treatment” procedure resembles the arrangement of PS II dimers in the native thylakoid membrane. (c) success of the treatment procedure was strongly dependent on the choice of the right starting detergent and the increase of the PEG MME concentration was accompanied by a parallel decrease of detergent concentration, facilitating the removal of the detergent belt. However, the water content of the channels within these PS II structures, compared with other high-resolution PSII structures, is not affected by the temperature the measurement was performed at or the degree of post-crystallization treatment that may vary based on the applied protocol (Hussein et al. 2021). In addition, Membrane Inlet Mass Spectrometry (MIMS) was used to compare the transition efficiency of PS II solution against treated PS II crystals, and no significant differences were found (Kern et al. 2018; Ibrahim et al. 2020; Hussein et al. 2021). Based on these observations, it is misleading to assume that PS II in the crystal packing after the “post-crystalization treatment” procedure is lacking water, especially within the channels, or is reduced in water oxidation activity, as was recently suggested (Sirohiwal and Pantazis 2022). On the contrary, when comparing the number of observed water molecules in several recent high-resolution structures obtained by cryo-EM and crystallography no indication for the presence of a lower number of waters in the PS II complex in crystalline “dehydrated” or “treated” samples versus untreated flash-frozen solution samples was evident: 1957, 1377 and 1000–1100 waters/monomer for the highest resolution “dehydrated/ treated” cryo XRD structure at 1.85 Å (Tanaka et al. 2017), 1.95 Å (Suga et al. 2015) and ~ 2 Å resolution (Ibrahim et al. 2020) respectively; and 1222 waters/monomer in the non-dehydrated cryo-EM structures at 1.95 Å resolution (Kato et al. 2021). From this comparison, it is clear that the number of modeled waters in a certain structure is a function of the obtained resolution, but is also dependent on the choice of refinement software. One additional important parameter is the measurement temperature, as higher temperatures lead to higher mobility and hence weaker localization of some waters in the structural data. By contrast, this comparison does not support the assertion that the”post-crystalliztion treatment” procedure affects the crystal water content within the channels of PS II.

## Comparison of channels

Based on the the available structures of PS II, we used Caver 3.0.3 (Chovancova et al. 2012) to investigate the channels that connect the Mn_4_CaO_5_ cluster to the lumenal surface in different oxygenic photosynthetic organisms, selecting one structure for each organism. Details are given in the legend of Table 3. The results obtained for PS II from mesophilic cyanobacteria, the red lineage, including red algae and diatoms, and the green lineage including green algae and higher plants are compared to those determined for the thermophilic cyanobacteria.

**Table 3:**
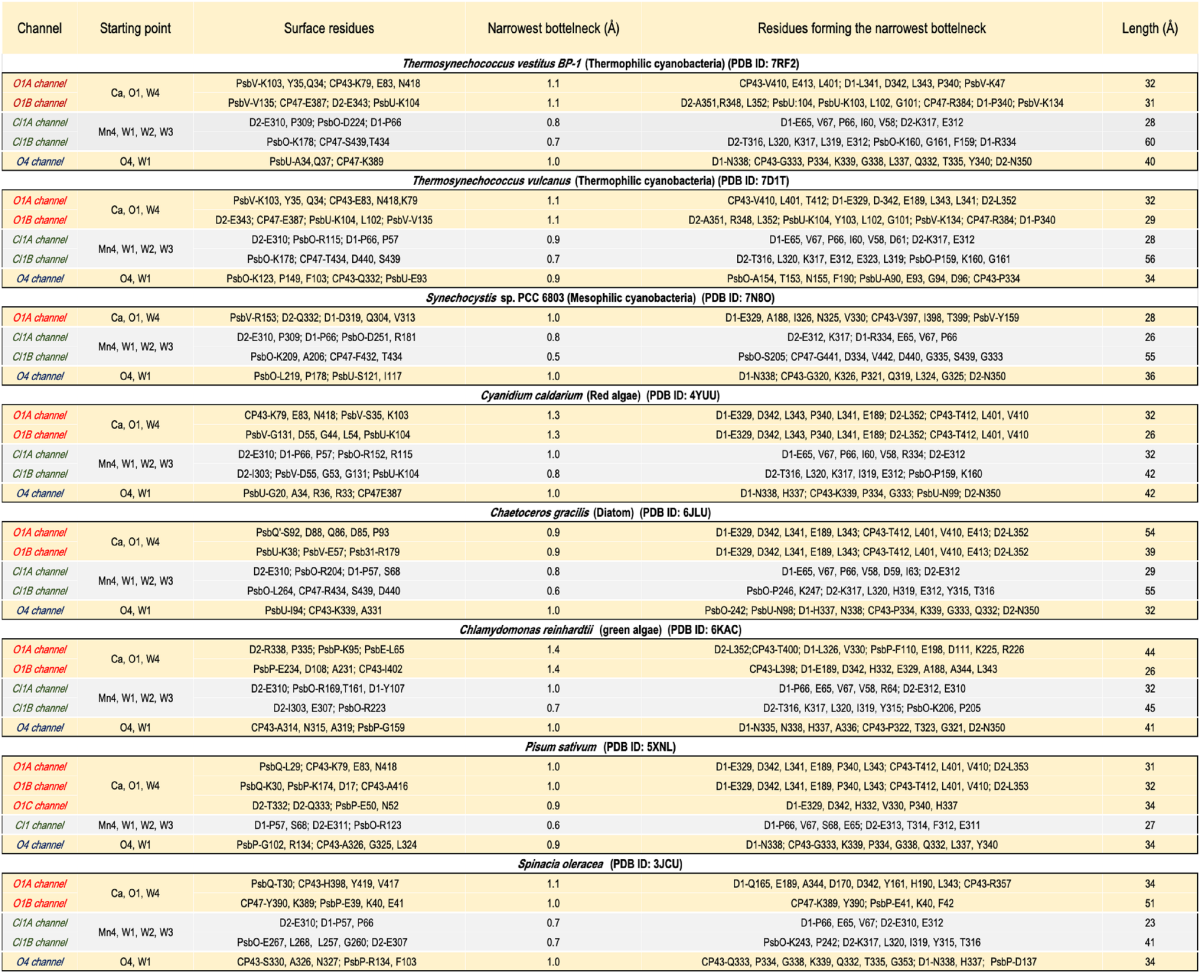
Characteristics of channels leading from the Mn_4_CaO_5_ cluster to the lumenal side of PS II (“surface” = at the bulk interface, endpoint of the channel) for the different organisms covered in this study

All the channel calculations were performed with the software version of CAVER 3.0 (Chovancova et al. 2012) (with shell radius and shell depth equal to 3.0 Å and 4.0 Å, respectively, the minimum probe radius used was 0.9 Å for all the calculations except for O4 channel calculation, where 0.5 Å was used). The starting point of the analysis is described above

The potential water channels currently proposed in the literature are mainly based on the crystal structures of the thermophilic cyanobacteria. The early studies in this area were performed by (Murray and Barber 2007a, b; Ho and Styring 2008; Ho 2008; Gabdulkhakov et al. 2009), based on searches for channels in the crystal structures. In addition to the direct crystal structure analysis, MD simulations were performed, e.g., to identify new channels, determine the water permeation energetics and study the water diffusion from the bulk (Vassiliev et al. 2010, 2012, 2013; Gabdulkhakov et al. 2015b; Capone et al. 2016; Sakashita et al. 2017a, 2020). These channels match some channels identified in the earlier crystal structures. The three main channels presently discussed are the Cl1 channel, the O1 channel, and the O4 channel (Table 3), and their corresponding names in other studies are summarized in Table 1.

### The Cl1 channel

**Fig. 3:**
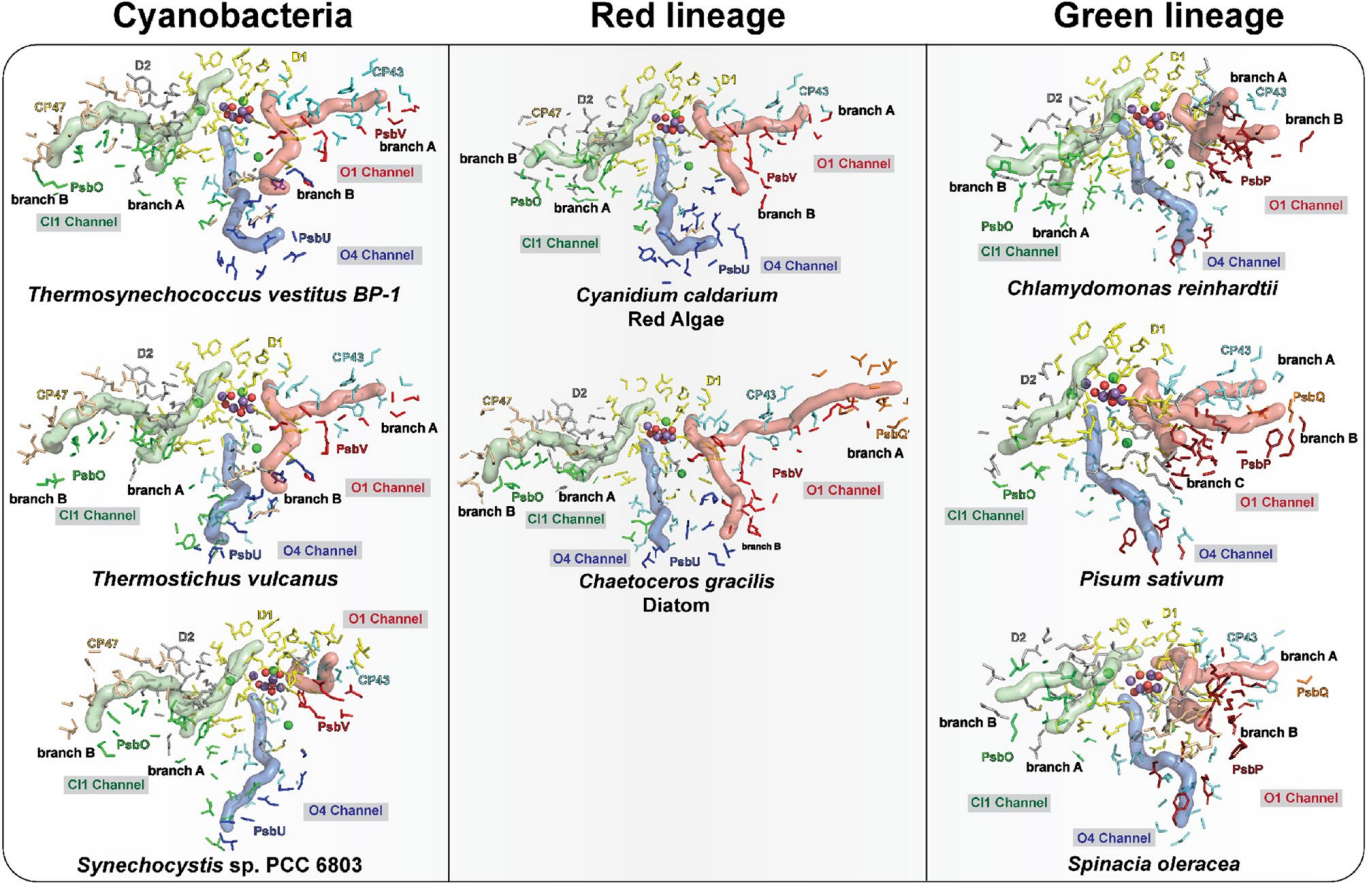
Possible water channels connecting the Mn_4_CaO_5_ cluster to the lumenal side of PS II from different oxygenic organisms. The channels were identified and analyzed using the CAVER program based on the PSII structures from *T. vestitus BP-1* (PDB:7RF2, RT-XFEL), *T. vulcanus* (PDB:7D1T, cryo-EM), *S.* sp. PCC 6803 (PDB:7N8O, cryo-EM), *C. caldarium* (PDB:4YUU, cryo-X-ray), *C. gracilis* (PDB: 6JLU, cryo-EM), *C. reinhardtii* (PDB:6KAC, cryo-EM), *P. sativum* (PDB:5XNL, cryo-EM), *S.oleracea* (PDB: 3JCU, cryo-EM). Residues lining the channels are shown in stick representation: D1 in yellow, D2 in gray, CP43 in cyan, CP47 in wheat, PsbO in green, PsbU in blue, PsbV in red, PsbQ’ and PsbQ in orange and PsbP in firebrick. Channels starting from Ca and O1 are shown in light red surface (O1 channel), from Mn4, W1, W2, and W3 and passing through Cl1 ion are shown in light green surface (Cl1 channel), and from O4 and W1 are shown in light blue surface (O4 channel). The Mn_4_CaO_5_ cluster and Cl ions are shown as spheres: Mn purple, Ca^2+^/Cl^−^ green, and O red

**Fig. 4:**
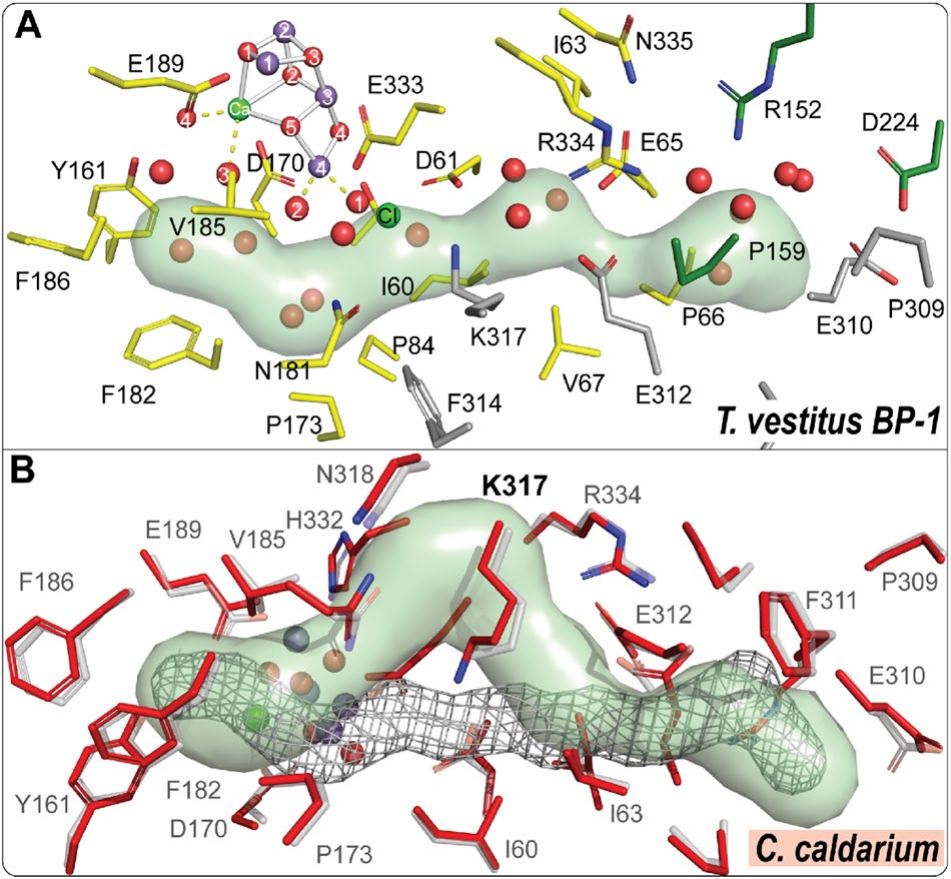
Cl1A channel and the surrounding environment. **A** Cl1A channel in *T. vestitus BP-1*. The channel is generated using the S_1_ state data (PDB:7RF2). Cl1A channel is in green. Amino acid residues from D1, D2, and PsbO subunits are colored yellow, gray, and green, respectively. **B** The calculated Cl1 channel in *C. caldarium* (shown in green) in comparison to the one in thermophilic cyanobacteria (shown as gray mesh). The structure of *C. caldarium* (PDB:4YUU) is shown in red and overlaid with the structure of *T. vestitus BP-1* (PDB:7RF2, shown in transparent gray)

In the thermophilic cyanobacteria, either in *T. vestitus BP-1*, or in *T. vulcanus,* the Cl1 Channel starts from the Mn4 side of the Mn_4_CaO_5_ cluster, passing along the Cl1 ion and connecting to the lumen through D1, D2, and PsbO domains for branch A, also known as "broad channel" or "channel 3", and D2, CP47 and PsbO for branch B (Fig. 3, Tables 1, 3). These subunits that participate in lining this channel are considered conserved among all the oxygenic photosynthetic organisms (Figs. 3, 4A).

**Fig. 5:**
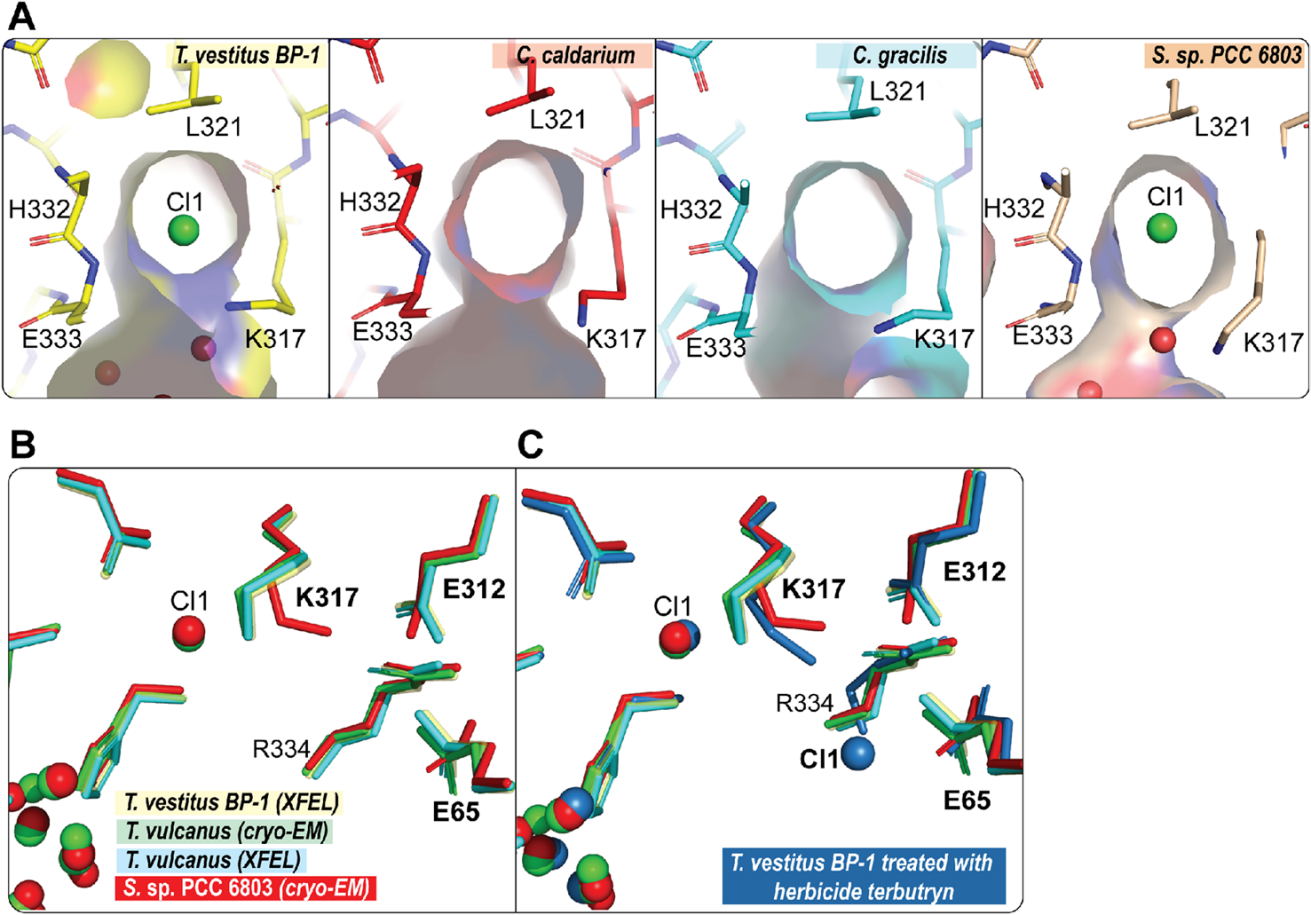
The position of Cl1 ion in different oxygenic photosynthesis organisms. **A** The binding pocket of the Cl1 ion in *C. caldarium* (PDB:4YUU), *C. gracilis* (PDB: 6JLU), and *S.* sp. PCC 6803 (PDB: 7N8O, cryo-EM) relative to *T. vestitus BP-1* (PDB:7RF2). Labels: Cl1 green, O red. **B**, **C** The changes in the position of Cl1 and the side chain of D2-K317 in *S.* sp. PCC 6803 (PDB: 7N8O, cryo-EM, in red) relative to *T. vestitus BP-1* (PDB:7RF2, XFEL, colored in yellow), *T. vulcanus* (PDB: 4UB6, XFEL, colored in cyan; PDB:7D1T, cryo-EM, colored in green) and *T. vestitus BP-1* treated with herbicide terbutryn (PDB:4V82, X-ray, colored in sky blue)

Surprisingly, the Cl1 ion was not modeled in the structures of the red algae *C. caldarium* and the diatom *C. gracilis.* However, we can see that the binding pocket of the Cl1 ion is maintained in these structures and is similar to the binding pocket of the Cl1 ion detected in the PS II structure in other organisms (see Fig. 5A). The absence of Cl1 in the structural model might be due to the limited resolution of these structures; 2.77 Å and 3.02 Å for *C. caldarium* (red algae) and *C. gracilis* (diatom), respectively (Table 2)*.* For example in the structure of *C. caldarium* (red algae), an apparent weak electron density appears in the corresponding location of the Cl1 which was modeled as a water molecule rather than a Cl^−^ ion. In the recent structure of *S.* sp. PCC 6803 (Gisriel et al. 2022), it was reported that for the dominant side chain conformation of D2-K317 the N^ζ^ atom is 5.1 Å away from the Cl1 ion position compared to an average distance of 3.1 Å in *T. vestitus BP-1* (PDB: 7RF2) and  *T. vulcanus* (PDB:4UB6, 7D1T) (Fig. 5B). Interestingly, we found that this position of D2-K317 is quite similar to the position of D2-K317 found in the structure obtained for herbicide (terbutryn) inhibited PS II from *T. vestitus BP-1* (Broser et al. 2011)(Fig. 5C). However, only in this inhibited PS II structure, another density for the Cl1 ion was found within 3.5 Å from the N^ζ^ atom of D2-K317 at 70% occupancy (Fig. 5C) and located more centrally within the Cl1 channel. The native Cl1 position was only 30% occupied in this structure, possibly indicating a structural flexibility of the D2-K317 side chain and the positioning of Cl1 depending on the conditions of the PS II complex and maybe the protonation state of the nearby residues D1-D61 and D1-E65 (Broser et al. 2011).

**Fig. 6:**
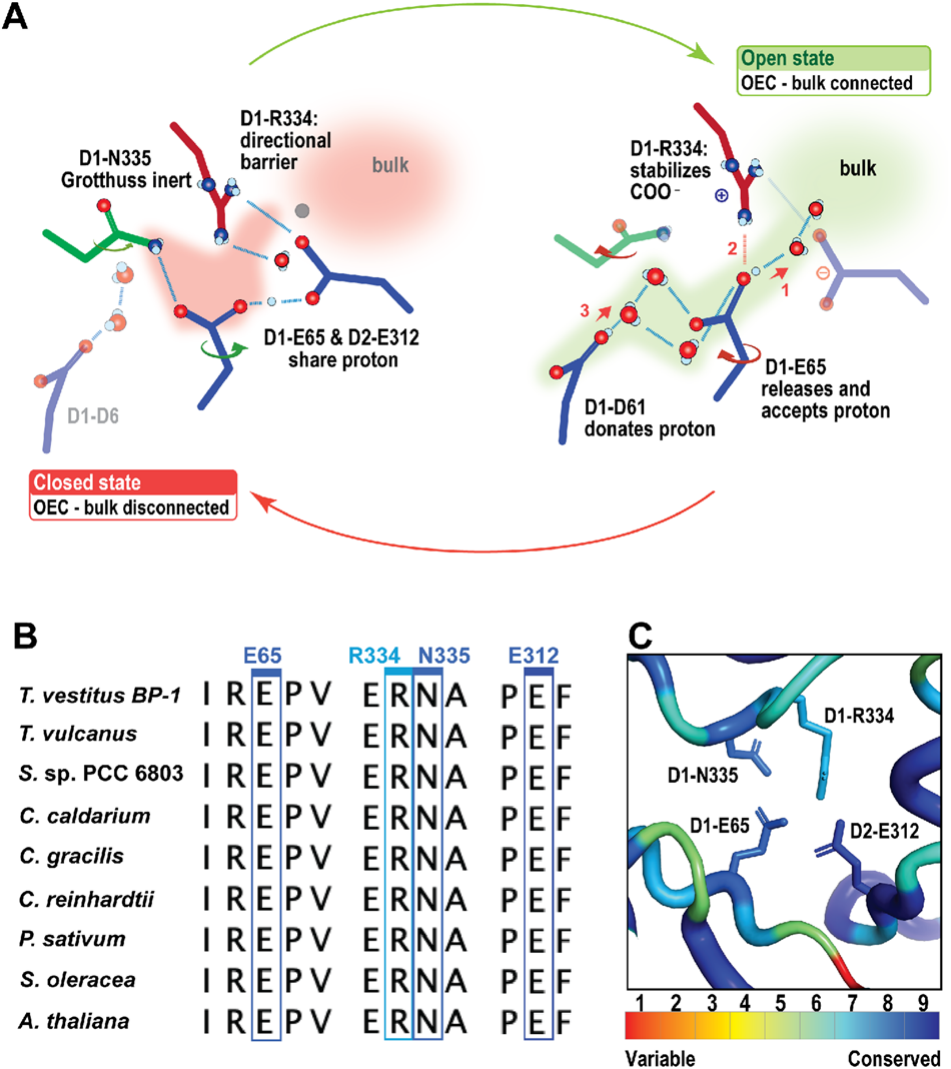
Proton gate residues in Cl1A channel. **A** The changes at the proton gate between the closed and open state. The H-bonding network in the closed state connecting the OEC to the bulk is disrupted, while it is well established in the open state (adapted from (Hussein et al. 2021)). **B** Partial sequence alignment of the proton gate residues D1-E65, D1-R334, D1-N334, and D2-E312 from *T. vestitus BP-1, T. vulcanus, S.* sp. PCC 6803*, C. caldarium, C. gracilis, C. reinhardtii, P. sativum, S. oleracea,* and *A. thaliana.* Sequence alignments were generated with NCBI-BLASTp. **C** ConSurf analysis (Ashkenazy et al. 2016) for the proton gate residues was performed for the D1 and D2 subunits. Coloring represents the degree of conservation across the identified homologs collected by UNIREF90 (Suzek et al. 2015) and clustered using the search algorithm HMMER (Mistry et al. 2013) (blue represents the highly conserved residues, and red is the most variable)

The Cl1A channel has been proposed previously as a proton release pathway during the S_2_ to S_3_ transition (Ishikita et al. 2006; Kuroda et al. 2021). A recent simulation study showed that the Cl1 channel is an energetically more favorable pathway for proton release toward the lumenal side than the other two channels, O1 or O4 (Kaur et al. 2021). Another mutational study combined with TRIR suggested that the proton release occurs mainly through the Cl1 channel during the S_3_ → S_0_ transition, but doesn't exclude the possibility of proton release through other paths during the S_2_ → S_3_ transition (Shimada et al. 2022). In our recent study of the S_2_ → S_3_ transition (Hussein et al. 2021), we observed structural changes around D1-E65, D2-E312 and D1-R334 associated with H-bond rearrangements along the channel, potentially related to the release of the proton from the cluster via the proton gate residues E65/E312 (Fig. 6A). Interestingly, we were able to locate a similar path in all the PS II structures using Caver. Only in the structure of *C. caldarium* (red algae) PS II the D1-K317 residue has a slightly different conformation that blocks the known path of that branch (Fig. 4B). Due to the limited resolution of the structure from *C. caldarium* (2.77 Å), we can not be confident about the side chain position of the D2-K317 residue. Nevertheless, another path was detected in this structure using Caver. This path shares the same start and end points as the Cl1A channel in thermophilic cyanobacteria (Fig. 4B & Table 3). Using partial sequence alignment between all studied species and the ConSurf server (Ashkenazy et al. 2016) to show the evolutionary conserved amino acid residues, we observed that this path, including the gate region is highly conserved (Fig. 6B, C).

### The O1 channel

The O1 Channel (Fig. 3), which is also known as "large channel" in thermophilic cyanobacteria, starts from the water (W3, W4) binding site on Ca and reaches the lumen at the interface of subunits D1, CP43, and PsbV for branch A and PsbU, PsbV, D2 and CP47 for branch B. This channel has been suggested to be the water intake pathway, at least during the water substrate binding during the S_2_ → S_3_ transition (Suga et al. 2019; Ibrahim et al. 2020; Hussein et al. 2021).

**Fig. 7:**
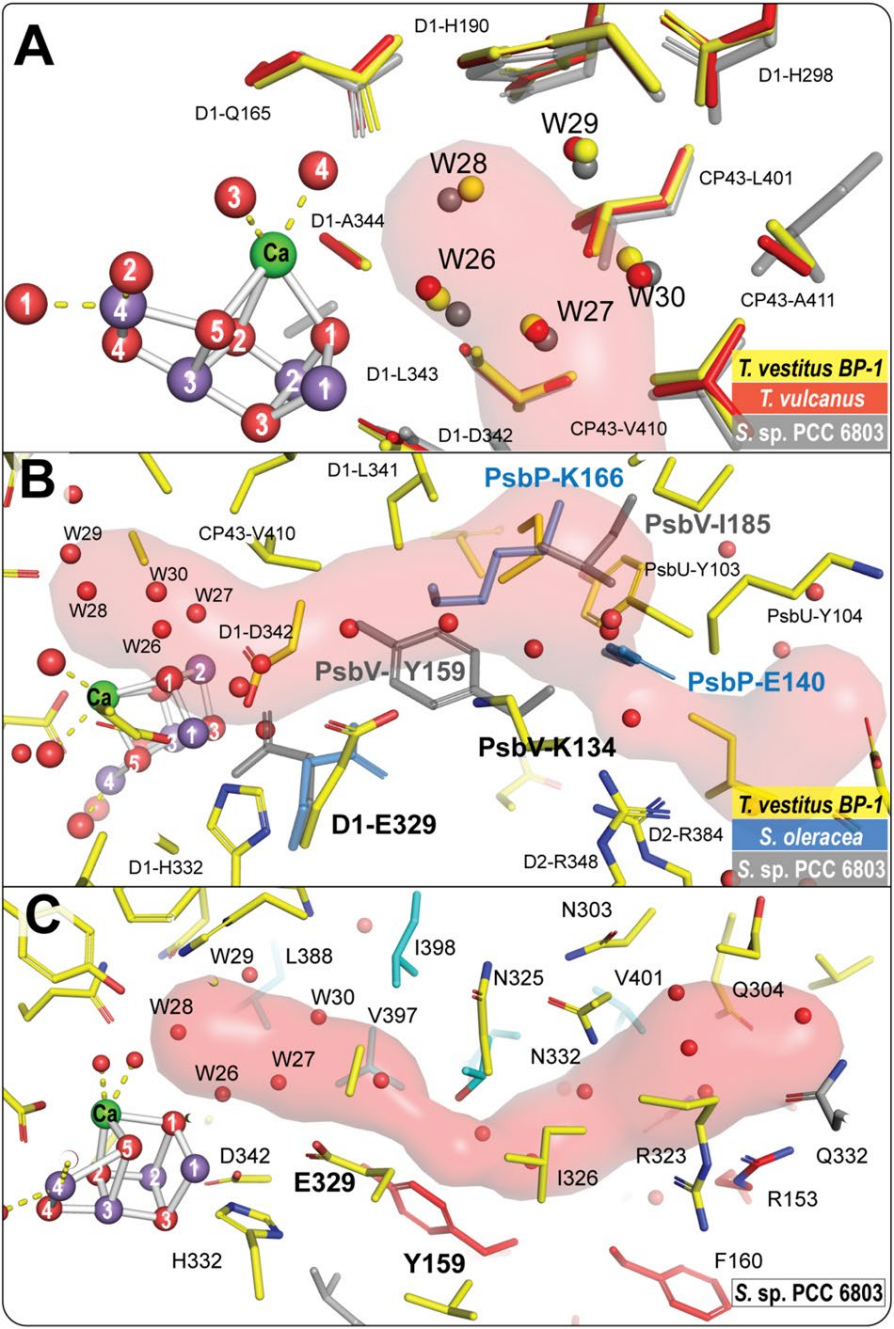
O1 channel in different photosynthetic organisms. **A** The conserved water wheel area (W26-27-28-29-30) at the beginning of the O1 channel. The figure shows a superposition of the structure of *T. vestitus BP-1* (PDB:7RF2), *T. vulcanus* (PDB: 4UB6), and the *S.* sp. PCC 6803 (PDB:7N8O, cryo-EM) colored in yellow, red and gray, respectively. **B** O1B channel in *T. vestitus BP-1* (PDB:7RF2) showing the residues that block that path in different organisms. Structures of *T. vestitus BP-1, S.* sp. PCC (PDB:7N8O), and *S. oleracea* are shown in yellow, gray and blue, respectively. **C** the O1 channel path in *S.* sp. 6803 (PDB:7N8O) generated by Caver (Chovancova et al. 2012). Amino acid residues from D1, D2, CP43, and PsbV subunits are colored yellow, gray, cyan, and red, respectively

Close to the cluster, the O1 channel is connected to the Mn_4_CaO_5_ via a network of five waters (W26-27-28-29-30) known as the ‘water wheel’ (Ibrahim et al. 2020). This water network is conserved among the high-resolution structures of PS II; *T. vestitus BP-1*, *T. vulcanus*, and *Synechocystis* sp. PCC 6803 (Fig. 7A). The beginning path of this channel is also found to be structurally conserved among all the studied structures (Fig. 3). However, since the ending of this channel goes through subunits PsbV and PsbU, the ending path of the channel should vary among the organisms (Table 3 and Figs. 2 & 3). In *S.* sp. PCC 6803*,* the amino acid residues Y159 and I185 of subunit PsbV block the known path of branch B of this channel detected in thermophilic cyanobacteria (Fig. 7B). Also, in the green lineage of the photosynthetic organisms, this path is blocked by either amino acid residues K225, D197, and E198 of subunit PsbP in *C. reinhardtti* or by amino acid residues K166 and E140 of subunit PsbP in *S. oleracea and P. sativium* (Fig. 7B). Nevertheless, our present comparison clearly reveals that one or more paths are still connecting the Mn_4_CaO_5_ cluster starting from the O1 and Ca sites via the water wheel with the lumen (Fig. 3 & Table 3). In this aspect, interestingly, the high-resolution (1.97 Å) cryo-EM structure of the *S.* sp. PCC 6803 in (Fig. 7C) exemplifies in more detail the conservation of the O1 channel connectivity. It is shown that the channel identified by Caver with narrowest bottelneck radius of 1 Å (Table 3), starting from the O1 side of the Mn_4_CaO_5_ cluster and reaching the lumen at the interface of subunits D1, D2, and PsbV is filled with water molecules, which is one of the essential features of water channels that could transfer water or protons.

**Fig. 8:**
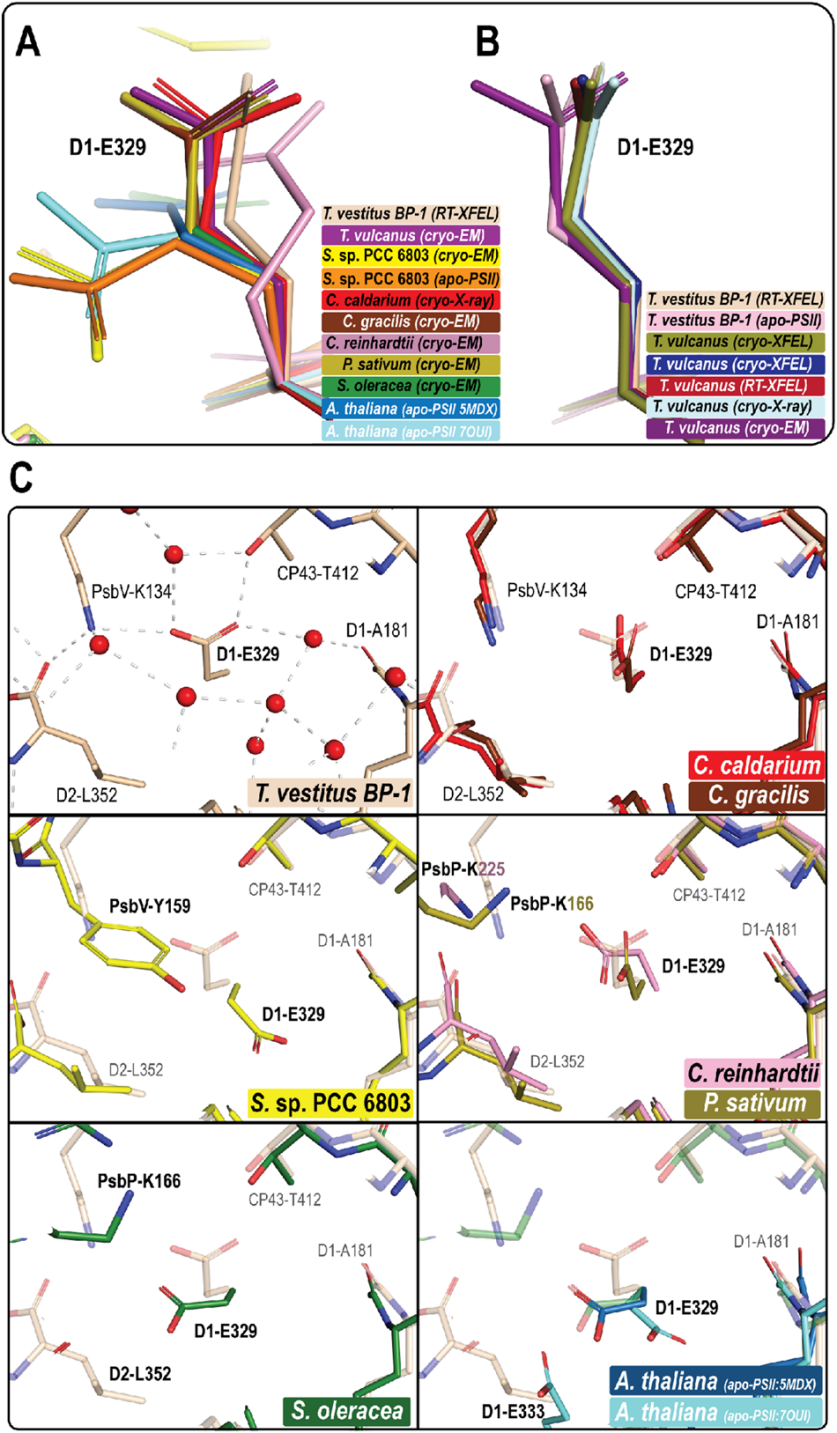
The conformation of the E329 side chain in different oxygenic photosynthesis organisms. **A** Superposition of different structures showing how the conformation of the E329 side chain varies; the structure of *T. vestitus BP-1* (PDB:7RF2, in wheat), *C. caldarium* (PDB:4YUU, in red), *C. gracilis* (PDB: 6JLU, in brown), *T. vulcanus* (PDB:7D1T, in deep purple), *P. sativum* (PDB:5XNL, in sand), *C. reinhardtii* (PDB:6KAC, in pink), *S. oleracea* (PDB: 3JCU, in green), *S.* sp. PCC 6803 (PDB:7N8O, in yellow), *S.* sp. PCC 6803 apo structure (PDB:6WJ6, in orange), *A. thaliana* apo structure (PDB: 5MDX, in sky blue), *A. thaliana* apo structure extracted with digitonin (PDB: 7OUI, in cyan). **B** The conformation of the E329 side chain in different structures of thermophilic cyanobacteria collected by different techniques; the RT-XFEL structure and the apo-PS II structure collected by cryo-X-ray from *T. vestitus BP-1* (PDB ID:7RF2 and PDB ID: 5MX2, respectively), five structures from *T. vulcanus, two* collected by *cryo-XFEL* (colored in sand (PDB: 6JLJ) and in blue (PDB: 4UB6)), and one each by RT-XFEL (PDB:5WS5), cryo-X-ray (PDB:3WU2) and cryo-EM (PDB:7D1T). **C** The surrounding environment at E329 among the different organisms. The environment of E329 in the thermophilic cyanobacteria (PDB:7RF2), the red algae *C. caldarium,* and the diatom *C. gracilis* is conserved, as shown in the first row. However, the environment is different in *S.* sp. PCC 6803 and also in all green lineage organisms. In *S.* sp. PCC 6803 (PDB:7N8O)*,* the position of PsbV-Y159 forces the E329 side chain to be in a different orientation compared to its position in thermophilic cyanobacteria. In the green linage organisms, the PsbV subunit is entirely replaced with the PsbP subunit, leaving the position of PsbV-K134 empty as in green algae *C. reinhardtii* (PDB:6KAC) and *P. sativum* (PDB: 5XNL). Moreover, the D2-L352 side chain is not conserved in spinach *S. oleracea* (PDB: 3JCU), which allows E329 to be in a different plane compared to its position in thermophilic cyanobacteria. For *A. thaliana*, there are two cryo-EM structures available for the apo-PSII; the position of the E329 side chain in one structure (PDB: 5MDX, colored in sky blue) is similar to the structure of spinach*.* However, in the other structure treated with digitonin (PDB: 7OUI, in cyan), the E329 side chain shows a different position which may be due to enhanced structural disorder in the D1 subunit

While the starting region of the O1 channel is structurally identical among the different species, the sidechain position of D1-E329 (Table 3), which participates in forming the first bottleneck of the channel, varies significantly (Fig. 8A). Our analysis shows that these differences could be due to several reasons; the first is the radiation damage that may happen during data collection, especially using cryo-EM. The comparison of the PS II structures from thermophilic cyanobacteria shows that the E329 side chain in the cryo-EM structure from *T. vulcanus* rotates significantly compared to the radiation-free structures, which were collected using XFEL, or the structures obtained using conventional synchrotron X-rays from *T. vulcanus* or *T. vestitus BP-1* (Fig. 8B)*.* Secondly, the orientation of the E329 headgroup may vary due to major structural differences in this region visible in *S.* sp. 6803 and all green lineage organisms as compared to thermophilic cyanobacteria (Fig. 8C). In *S.* sp. 6803, the location of PsbV-Y159 forces the E329 side chain to be in a different orientation, whereas, in the green lineage organisms, the PsbV subunit is entirely replaced with the PsbP subunit, leaving the position of PsbV-K134, which is near E329, empty (Figs. 7B and 8C). Moreover, the D2-L352 residue in the direct vicinity of E329 is not conserved in spinach *S. oleracea* (PDB: 3JCU), which gives E329 more freedom and allows it to be in a different plane compared to its position in thermophilic cyanobacteria (Fig. 8C). Thirdly, E329 may be affected by structural changes in the D1 subunit induced by the sample preparation, as observed in the apo-PSII from *T. vestitus BP-1* (Zhang et al. 2017), due to the harsh treatment used to remove the Mn cluster (Fig. 8B), and also in the PS II structure in *A. thaliana* extracted with digitonin (Fig. 8C) (Graca et al. 2021).

### The O4 channel

**Fig. 9:**
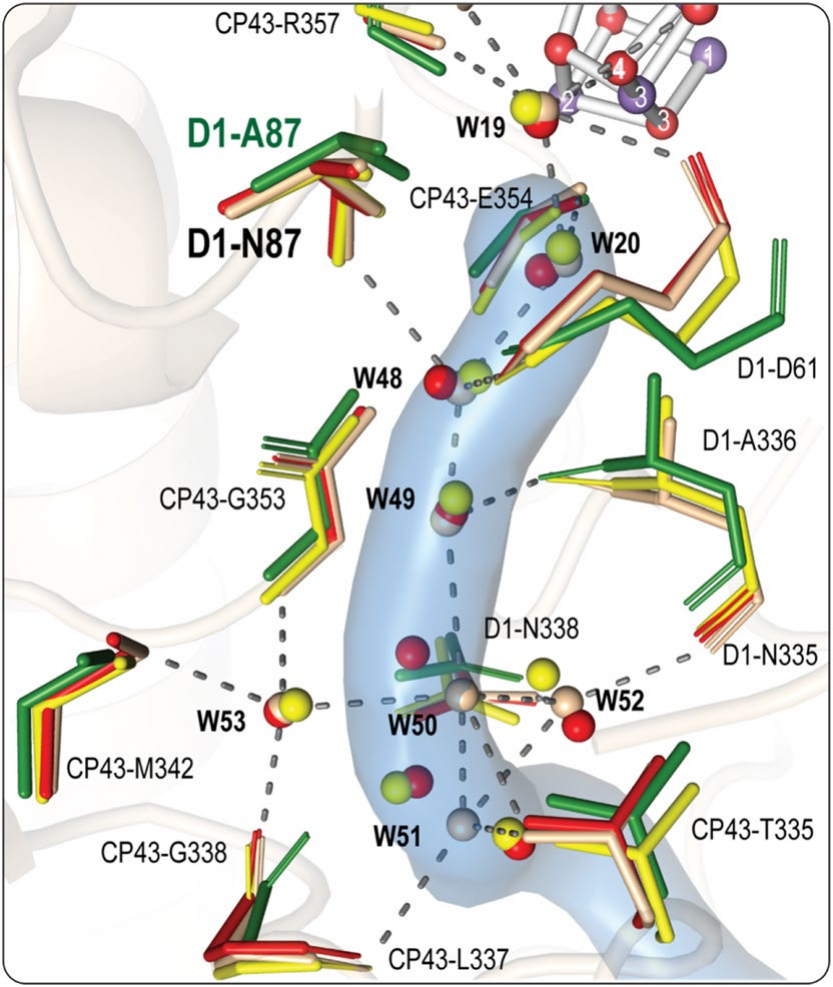
Conserved residues and water molecules in the O4 channel. The water molecules and the residues lining the channel shown are from *T. vestitus BP-1*(PDB: 7RF2, S_1_ state, in wheat), *T. vulcanus* (PDB:7D1T, in red), *S.* sp. PCC 6803 (PDB:7N8O, in yellow) and *S. oleracea* (PDB: 3JCU, in green). The O4 channel from *T. vestitus BP-1* is shown as a light blue surface

The third channel determined in thermophilic cyanobacteria is the O4 channel, also known as the narrow channel. In thermophilic cyanobacteria, this channel starts near the O4 atom of the cluster, extending through D1, CP43, and D2 subunits before exiting PS II through a cavity formed by subunits PsbO and PsbU (Fig. 3). Our investigations show that this channel proceeds through the same path in all studied species (Fig. 3), which is expected since the D1, CP43, and D2 subunits are conserved subunits. However, the later parts of the O4 channel near the lumenal surfaces show different orientations. In the cyanobacteria and the red lineage organisms that contain the PsbU subunit, a subtle change in the path at the end of the identified O4 channel is likely due to the higher positional mobility of the side chains near the lumen. However, the more drastic change is observed in the green lineage organisms where the PsbU subunit is replaced by PsbP instead as the exit path. Another significant difference observed only in the structures of PS II in higher plants is the replacement of residue D1-N87 with D1-A87 in the beginning of the channel (Fig. 9).

The O4 channel harbors the longest non-disrupted H-bonded water chain identified in thermophilic cyanobacteria's dark-adapted PS II. Several studies have suggested this channel to be ideal for non-rate-limiting proton release. Our analysis of all high-resolution structures shows that almost all the water molecules present along the first 15 Å of the channel are conserved among cyanobacteria (Fig. 9). Moreover, the simulation study by Ishikita and colleagues (Sakashita et al. 2017b) shows that the equivalent water molecules are also present in PS II of spinach*.* Interestingly*,* the second water in this chain, W20, disappears during the S_2_ → S_3_ → S_0_ transitions, and is only restored after the formation of the S_0_-state, resulting in disrupting the H-bonded network and hence the proton release via the O4 channel (Kern et al. 2018; Suga et al. 2019). Therefore, the O4 channel is likely the proton release pathway only during the S_0_ → S_1_ transition. On the other hand, the substitution of the bulkier Asparagine for Alanine at the D1-87 position in higher plants results in enlarging the channel's beginning which may allow and facilitate the transport of water and water analogs like methanol, as observed for spinach (Oyala et al. 2014; Retegan and Pantazis 2017) and in a D1-N87A mutant of *Synechocystis* sp. PCC 6803 (Kalendra et al. 2022). Based on this observation, it was suggested that this channel could possibly be involved in substrate water delivery in higher plants (Retegan and Pantazis 2017).

## Summary and outlook

The remarkable advances in the techniques used for characterization of PS II in the last decade has enabled us to visualize the structures of PS II from different organisms, and in some cases, under physiological temperatures. The Cl1 channel, including its proton release gate, is highly conserved among all photosynthetic species and thus appears to be critical for proton release in the S_2_ → S_3_ and S_3_ → S_0_ transitions. The O1 channel varies most between species, which includes the D1-E329 side chain orientation. However, in all organisms at least one branch extends all the way from Ca/O1 via the ‘water wheel’ region to the lumen. Thus, the comparison is consistent with the O1 channels functioning as a water delivery path. We propose that the exact shape of the channel leading from the ‘water wheel’ to the lumen is less critical for water access. The inner part of the O4 channel is, like the entire Cl1 channel, highly conserved among all species, but some variation is observed in the lumenal region. It has been previously suggested to function in either proton release or as water access channel. Our comparison does not provide a unique answer to this question. However, the highly ordered water chain that has an S-state dependent/gated connection to the Mn_4_CaO_5_ cluster via the water molecule (W19) directly bound to O4 (Fig. 9) may indicate that it functions in proton release during the S_0_ → S_1_ transition (Kern et al. 2018; Suga et al. 2019).

Information obtained from comparing PS II structures from different photosynthetic organisms helps us understand what the fundamentally important part in the architecture of PS II for the light-driven water oxidation reaction in nature is, and to what extent PS II tolerates structural variability. Yet, we need to be attentive to distinguish intrinsic structural properties from extrinsic effects that arise from differences in experimental conditions. It requires interpreting data from various methods, while considering the uncertainties, advantages and shortcomings that are unique to each method. In any respect, we are at a major step towards understanding the chemistry of the water oxidation reaction, not only the mechanism of the catalytic center, but also recognizing how the protein and water network function to direct complicated multielectron/multiproton reactions for high selectivity and efficiency of catalysis. Understanding such interplay between the catalytic metal center and its environment will provide inspiration for how to control reactions in artificial photosynthetic systems.

## Data Availability

The detailed results of the performed analysis are freely available from the authors upon request.
